# Physical activity as a possible mechanism behind the relationship between green space and health: A multilevel analysis

**DOI:** 10.1186/1471-2458-8-206

**Published:** 2008-06-10

**Authors:** Jolanda Maas, Robert A Verheij, Peter Spreeuwenberg, Peter P Groenewegen

**Affiliations:** 1 NIVEL (Netherlands Institute for Health Services Research), PO Box 1568, 3500 BN, Utrecht, The Netherlands; 2 Utrecht University, Department of Human Geography, Department of Sociology, Utrecht, The Netherlands

## Abstract

**Background:**

The aim of this study was to investigate whether physical activity (in general, and more specifically, walking and cycling during leisure time and for commuting purposes, sports and gardening) is an underlying mechanism in the relationship between the amount of green space in people's direct living environment and self-perceived health. To study this, we first investigated whether the amount of green space in the living environment is related to the level of physical activity. When an association between green space and physical activity was found, we analysed whether this could explain the relationship between green space and health.

**Methods:**

The study includes 4.899 Dutch people who were interviewed about physical activity, self-perceived health and demographic and socioeconomic background. The amount of green space within a one-kilometre and a three-kilometre radius around the postal code coordinates was calculated for each individual. Multivariate multilevel analyses and multilevel logistic regression analyses were performed at two levels and with controls for socio-demographic characteristics and urbanicity.

**Results:**

No relationship was found between the amount of green space in the living environment and whether or not people meet the Dutch public health recommendations for physical activity, sports and walking for commuting purposes. People with more green space in their living environment walked and cycled less often and fewer minutes during leisure time; people with more green space garden more often and spend more time on gardening. Furthermore, if people cycle for commuting purposes they spend more time on this if they live in a greener living environment. Whether or not people garden, the time spent on gardening and time spent on cycling for commuting purposes did not explain the relationship between green space and health.

**Conclusion:**

Our study indicates that the amount of green space in the living environment is scarcely related to the level of physical activity. Furthermore, the amount of physical activity undertaken in greener living environments does not explain the relationship between green space and health.

## Background

There is increasing attention and evidence for a positive relation between the amount of green space in the living environment and people's health and well-being. Several studies have shown that a more natural living environment positively influences people's self-perceived health and leads to lower mortality risks (e.g. [1-3]). However, little is known about the way in which green space exerts a beneficial effect on health. Several mechanisms may be underlying, of which the following are most commonly mentioned: recovery from stress and attention fatigue, encouragement of physical activity and facilitation of social contact [4,5]. A large number of mainly experimental studies have produced strong evidence of the positive effect of nature on recovery from stress and attention fatigue [5,6]. Less is known about other possible underlying mechanisms, such as physical activity. In this study we aim to investigate whether physical activity is a possible mechanism behind the relationship between green space and health.

It has long been known that being physically active has positive health effects [7]. If a green living environment provides an incentive to be physically active, this could positively influence people's health. Literature shows that people are inclined to undertake physical activity in aesthetically appealing environments [8-11]. Natural environments are perceived to be more aesthetically appealing than built-up environments [12,13]. Therefore, natural environments may stimulate people to undertake healthy physical activities, such as walking or cycling, or to choose these activities as a mode of transport, and to spend more time on them [14,15].

Because of increasing urbanisation, combined with a spatial planning policy of densification, more people face the prospect of living in residential environments with fewer green resources. If the amount of green space in the living environment stimulates people to be physically active, the reduction of green space could have consequences for the amount of physical activity of the population.

Most of the literature concerning the relation between green in the living environment and the amount of physical activity focuses on specific types of physical activity, namely walking and cycling. Walking and cycling have been placed firmly on the public health agenda as a result of the awareness that health benefits could be derived by engaging in 30 minutes of moderate exercise every day [16]. With regard to the influence of green space, a review of environmental influences on walking behaviour concludes that the aesthetic nature of the environment and accessibility of destinations, like parks and beaches, encourage walking [17]. Pikora et al. (2003) [9] concluded on the basis of available literature that attractiveness of the streetscape was one of the most important features related to walking and cycling. An attractive streetscape included, among other things, trees, wide grassy verges, parks, private gardens, diverse and interesting natural sights. These reviews mainly focused on research performed in Australia, the United States and the United Kingdom. Only a few studies have been performed on the relationship between the general level of physical activity and green space. A study by Ellaway et al. (2005) [18] which used data collected in eight European countries showed that "for respondents whose residential environment contains high levels of greenery, the likelihood of being more physically active is more than three times as high, and the likelihood of being overweight and obese is about 40% less". However, on the other hand, a study by Hillsdon et al. (2006) [19], conducted in the U.K., found no association between hours per week of recreational physical activity and access to and quality of any urban green spaces in a cohort of middle-aged adults.

Hoehner et al. (2005) [20] and McGinn et al. (2007) [21] both investigated the correlation between the presence of trees along the neighbourhood streets and physical activity. McGinn et al. found that those who perceived that lack of trees for shade was not a problem, or a barrier to physical activity were more likely to be active during leisure time physical activity [21]. Hoehner et al., however, found no association between trees along the neighbourhood streets and transportation or recreational activity [20].

In the Netherlands, little support is found for a positive relationship between the amount of local green space and walking and cycling [22-24]. Studies performed in the Netherlands show that the availability of local green space has little or no influence on how often people walk or cycle. In the case of poor availability of green space, people seem to walk and cycle more often in a built-up environment. For cycling, trips in the green environment tend to be longer than trips in the built-up environment [22,23]. A study by Wendel-Vos et al. (2004) [24] showed that the amount of green and recreational space, specifically sports grounds and parks, within a radius of 300 and 500 meters of the participants' homes was positively associated with time spent on bicycling in a Dutch city. According to Wendel-Vos et al. (2004) [24], it is however likely that this reflects the fact that people living in the outskirts of towns spend more time bicycling to the city. A study by Den Hertog et al. (2006) [25], performed in four different districts in Amsterdam, showed that a park of good quality in the district stimulates active behaviour, especially for children. However, people in the more urban neighbourhoods – neighbourhoods with less green space – appeared to be more physically active and were less often overweight. This was attributed to the design of the more urban districts, which had more facilities at walking or cycling distance and had no private parking space.

Besides the fact that a green environment can invite people to be physically active, it might also encourage people to exercise for longer periods of time. Research by Pennebaker & Lightner (1980) [26] showed that joggers who jogged in a green stimulating environment were distracted from signals of fatigue and physical symptoms. Furthermore, research by Pretty et al. (2007) [27] showed that people who participated in outdoor exercise programmes more often complete the programme than people who participated in indoor exercise programmes. These two studies imply that people engage in physical activity for longer periods in a green environment than in an indoor environment.

Overall, the available studies indicate that the evidence for a relationship between the amount of green space and the level of physical activity is limited. There are only indications for a positive relationship between an attractive streetscape and the amount of walking and cycling in Australia, the United States and the U.K. For other forms of physical activity and in other countries the available research is lacking or inconclusive. Furthermore, none of the described studies also link the possibly higher level of physical activity with people's health condition to see if the level of physical activity may be a mechanism underlying the relationship between green space and health.

It is interesting to investigate whether there is an association between green space and the level of physical activity particularly in the Netherlands, because of its strong walking and cycling culture in combination with its high degree of urbanisation.

The aim of this study was to investigate whether the relationship between green space and physical activity can be an underlying mechanism in the relationship between green space and self-perceived health in the Netherlands.

More specifically, the following research questions will be addressed:

1. Do people with a greener living environment more often meet the Dutch public health recommendations for physical activity?

2. Are people with a greener living environment more often physically active and do they spend more time on physical activity?

3. Can the amount of physical activity undertaken in greener living environments explain (part of) the relationship between green space and health?

Different types of physical activity which can take place in green areas will be considered. First of all, we will investigate whether people with more green space in their living environment more often meet the Dutch public health recommendations for physical activity, which states that people should engage in at least 30 minutes of moderate-intensity physical activity on at least 5 days per week [28]. Furthermore, the following types of physical activity will be considered which can be conducted directly from the home and can be influenced by the amount of green space: walking and cycling (both during leisure time as well as for commuting purposes), sports (for instance running, inline-skating) and gardening.

The relationship will be analysed for different types of green space to discover which type of natural surroundings particularly promote the level of physical activity. Furthermore, the relationship is analysed for different levels of urbanicity. Urban areas are often characterised by limited green space and a high availability of facilities (e.g. shops, services) at walking and cycling distance. On the other hand, in rural areas there is lots of green space, but people often have to use the car to visit facilities. Finally, the relationship will be analysed for different age groups and different socio-economic groups, because it is hypothesized that the correlation is likely to be stronger for groups that spend more time in the vicinity of their homes: youth and the elderly and people with a lower socio-economic status [15].

## Methods

### Population

The data were derived from two different datasets that were combined for this study. The data concerning health and physical activity originate from the Second Dutch National Survey of General Practice (DNSGP-2)[29]. Data for the DNSGP-2 were gathered in 2001 via 104 general practices. A random sample of the practice population (n = 5.265) were interviewed about topics including their self-reported health status, their level of physical activity and their demographic and socio-economic background characteristics. The important epidemiological criterion of covering the whole population at risk is met, since almost all non-institutionalised Dutch citizens are registered with a general practice [29]. Privacy of the participating persons is guaranteed, which is in accordance with Dutch legislation, and the study was approved by the Dutch Data Protection Authority. Patients were informed about the study prior to data collection and had the opportunity to opt out [29].

Environmental data were derived from the National Land Cover Classification database (LGN4), which contains the dominant type of land use of each 25 × 25 meter grid cell in the whole of the Netherlands in 2001 [30]. The two datasets were matched on the basis of x and y coordinates of the respondent's six character postal code (the same six character postal code is shared by no more than about 15 to 20 households). The percentage of green space within a 1-km radius as well as within a 3-km radius was calculated around these coordinates.

Only respondents who had valid responses on all relevant variables were included, leaving 4.899 respondents for inclusion.

### Perceived general health

Perceived general health was self rated by respondents by replying to the following statement: "In general, would you say that your health is...". They could respond by one of the following categories: excellent/very good/good/moderate/bad. The scores were dichotomised with the scores 'excellent', 'very good' or 'good' classified as healthy. This kind of operationalisation has been shown to be valid and predictive of health indicators in numerous studies [31,32].

### Physical activity

Level of physical activity was assessed using the short questionnaire to assess health enhancing physical activity (in short: SQUASH). The SQUASH is 'a fairly reliable and reasonably valid questionnaire' which 'may be used to order subjects according to their level of physical activity in an adult population' [33]. The questionnaire was completed by people aged above 12 and the interviews were spread over a whole year (to avoid seasonal differences). The questionnaire includes questions on four domains of physical activity, viz. commuting activities (walking and bicycling), occupational physical activity, household activity, and leisure-time physical activity (walking, bicycling, gardening and sports). Three main queries were asked: days per week, average time per day and intensity.

For this study, only commuting activities and leisure-time physical activity were taken into account, because it is expected that occupational and household activity are not influenced by the amount of green space in the living environment.

From the SQUASH the total number of minutes of walking, cycling (both during leisure time and commuting purposes), sport activities and gardening per week were calculated by multiplying the number of days per week spent on the activities with the number of minutes per day spent on the activity. Furthermore, dummy variables were created which stated whether or not people spent time on the different physical activities and whether or not people were physically active for 30 minutes on at least five days per week.

In our analysis of walking and cycling for commuting purposes, we only included those who had a job or went to school (2.816 respondents). In the analysis for gardening, we only included those who had a garden (3.951 respondents).

### Characteristics of respondents' living environment

Information on the environmental characteristics was derived from the LGN4 database. The LGN database distinguishes 39 land use classes including crop types, forest types, water, various urban classes and semi-natural classes [30,34]. The total percentage of green space in the respondents' living environment was measured within a 1-km radius and within a 3-km radius around a respondent's home, to see whether green space close by has a stronger or weaker effect than green space further away. A 1-km (equals 12 minutes walking) and a 3-km (equals 12 minutes cycling) radius were chosen because these distances could be easily undertaken from people's home. Only green spaces that have a dominant position in the 25 by 25 meter grid cell will be regarded as green space in the dataset. Gardens and small-scale green spaces, such as roadside trees and grassy verges are not regarded as green space in our study if they had no dominant position in the grid cell.

The total percentage of green space includes all urban green space, agricultural green space, forests and nature conservation areas. To discover which types of natural surroundings particularly promote the level of physical activity, we calculated the percentages of the following categories inside both a 1-km and a 3-km radius, the percentage of agricultural green space, the percentage of natural green space (forests, peat grassland, etc.), and the percentage of urban green space (woods and grassy areas in built-up environments).

### Urbanicity

Another environmental characteristic is urbanicity. This variable consists of five categories ranging from very strongly urban (1) to non-urban (5), and was measured at municipal level. The indicator is based on the number of households per square km and is widely used in the Netherlands [35].

### Demographic and socio-economic characteristics

Individual characteristics such as age, gender and socio-economic status, also play an important role in determining the level of physical activity [36-38]. Furthermore, it is important to realise that part of the relationship between green space and physical activity may be the result of direct or indirect selection. Direct selection takes place when people who like to be physically active in green spaces have a higher chance of living in a green environment, and people who feel healthy more often engage in physical activity. Indirect selection takes place when people with certain characteristics related to higher levels of physical activity (such as socio-economic status) can afford to live in a favourable environment. Migration flows are related to such socio-demographic characteristics as age, income and education [39].

To rule out selection effects as much as possible in a cross-sectional survey we took several demographic and socio-economic characteristics into account.

The demographic characteristics taken into account were gender (female = 1) and age (in years). For the analyses concerning the relationship between the percentage of green space in the living environment and the level of physical activity, age was divided into five categories (viz. children (aged 12–17 years); youth (aged 18–25 years); young adults (aged 26–40 years); older adults (aged 41–65 years), elderly (aged 65+)), because there was a non-linear relationship between age and the different forms of physical activity.

Socio-economic status was measured by the level of education (low, middle, high) and household income (high income (net monthly income > 2.450 euros), middle income (net monthly income between 1.350 and 2.450 euros) and low income (net monthly income < 1.350 euros)), which were also categorised because there was a non-linear relationship. In the analysis of the relationship between the general level of physical activity and the amount of green space, we also included a dummy variable indicating whether or not people had a garden.

### Statistical analyses

To study the relationship between the amount of green space and different types of physical activity we used a multivariate multilevel model, controlling for demographic and socio-economic characteristics and urbanicity. In a multivariate multilevel model two dependent variables can be included in one model, and the outcomes can be studied simultaneously. A multilevel logistic regression analysis was used to find out whether people with more green in their living environment have a higher chance of being physically active. A Poisson model was used to analyse the relationship between the amount of green space in the living environment and the number of minutes spent on (specific forms of) physical activity, because the responses were not normally distributed. We included two levels, viz. individuals and practices, because of the two-stage sampling design within DNSGP-2. Multilevel logistic regression analyses were performed to investigate the relationship between the amount of green space in the living environment and whether or not people meet the public health recommendations for physical activity.

Because we wanted to compare the relationship between different levels of urbanicity and different age and socio-economic subgroups we used interaction effects between the level of urbanicity or subgroup variable and the green indicator. Because of small numbers in the subgroups when looking at the duration of activity, we did not analyse differences in duration for age groups.

When we found a significant positive relationship between the amount of green space and physical activity we conducted a multilevel logistic regression analysis to analyse whether the found relationship could explain the relationship between green space and health. In all analyses we controlled for demographic characteristics, socioeconomic background characteristics and urbanicity. The multilevel analyses were performed with MLwiN.

## Results

Before analysing the relationship between the amount of green space in people's living environment and their level of physical activity, we looked at the bivariate correlation between the percentage of green space and degree of urbanicity. Urbanicity (high-low) was strongly positively related to the total percentage of green space (r = .60). Concerning the different types of green space, it was strongly positively related to the percentage of agricultural green space (r = .64) and was negatively related to the percentage of urban green space (r = -.42). The correlation with the amount of natural green space was much smaller (r = .25). This indicates that agricultural green areas dominate the total amount of green space (Table 1).

Besides the characteristics of the study population, Table 2 shows that 51.7% of the study population meet the public health recommendations for healthy physical activity. Walking and cycling during leisure time are the activities that are undertaken by the largest part of the population. Relatively few people walk for commuting purposes.

**Table 1:** Mean (standard deviation) of the percentage of green space in a 1-km and a 3-km radius around people's home in different levels of urbanicity

	**Very highly urban areas (n= 842)**	**Highly urban areas (n = 915)**	**Moderately urban areas (n = 963)**	**Slightly urban areas (n = 1.286)**	**Non urban areas (n = 893)**
**1 km**					
% total green	25.8 (17.3)	27.5 (16.5)	36.6 (19.3)	49.3 (21.3)	68.2 (17.6)
% agricultural green	6.8 (13.0)	8.3 (13.1)	20.7 (18.6)	32.4 (25.0)	56.6 (19.7)
% natural green	0.4 (1.8)	3.2 (6.7)	1.5 (3.7)	5.2 (7.4)	5.0 (7.6)
% urban green	18.6 (11.7)	16.0 (7.7)	14.4 (8.5)	11.7 (7.2)	6.6 (4.8)
					
**3 km**					
% total green	36.2 (16.4)	45.6 (13.2)	58.8 (15.2)	71.7 (13.2)	82.7 (12.2)
% agricultural green	17.0 (16.5)	23.5 (14.3)	43.0 (16.4)	55.7 (17.7)	68.4 (12.8)
% natural green	1.4 (2.2)	6.6 (8.8)	5.3 (5.0)	8.0 (6.0)	11.0 (8.9)
% urban green	17.8 (6.5)	15.5 (5.0)	10.5 (5.9)	8.0 (5.4)	3.3 (2.2)

**Table 2:** Percentual distribution of characteristics of the study population (n = 4.899)

	**Characteristics of the respondents**
**Demographic characteristics**	
**Gender**	
Female	54.4%
Male	45.6%
	
**Age**	
Child/adolescent (12–17 year)	7.8%
Youth (18–25 year)	6.9%
Young adults (26–40 year)	33.7%
Older adults (41–65 year)	33.6%
Elderly (>65 year)	18.1%
	
**Socio-economic characteristics**	
**Level of education**	
Low	20.6%
Middle	59.8%
High	19.7%
	
**Income**	
Low	32%
Middle	44.1%
High	23.8%
	
**Other characteristics**	
% of people with a garden	80.6%
	
**Urbanicity**	
Very highly urban	17.2%
Highly urban	18.7%
Moderately urban	19.7%
Sligthly urban	26.3%
Non urban	18.2%
	
**Physical Activity**	
% meets the Dutch public health recommendations for physical activity	51.7%
% of people actively engaged in sports activities	44.6%
Average (sd) number of minutes spent on sports activities per week	209 (236)
% of people who walk during leisure time	60.4%
Average (sd) number of minutes spent on walking during leisure time per week	214 (229)
% of people who cycle during leisure time	54.5%
Average (sd) number of minutes spent on cycling during leisure time per week	186 (199)
% of people who walk for commuting purposes	8.2%
Average (sd) number of minutes spent on walking for commuting purposes per week	146 (177)
% of people who cycle for commuting purposes	27.7%
Average (sd) number of minutes spent on cycling for commuting purposes per week	136 (123)
% of people who garden	39.7%
Average (sd) number of minutes spent on gardening per week	224 (279)
	
**Health**	
Percentage with perceived general health 'good', 'very good' or 'excellent'	82.2%

### Meeting public health recommendations for physical activity

Table 3 shows the result for the logistic multilevel analyses on the relationship between the percentage of green space in the living environment and whether or not people meet the Dutch public health recommendations for healthy physical activity. Models 1 and 2 show that there is no significant relationship between the percentage of green space and meeting the public health recommendations for physical activity, when controlling for demographic and socio-economic characteristics of the individual and urbanicity. People with more green space in their living environment do not more often meet the public health recommendations for physical activity.

**Table 3:** Multilevel logistic regression analysis of the influence of green space on whether or not people meet the public health recommendations for physical activity: parameter and standard error [p-value] (n = 4.899)

	**Meeting public health recommendations for physical activity (yes = 1)**	
	**1 km**	**3 km**

Percentage of green (1 km)	-.0004 (.002) [p = .808]	
Percentage of green (3 km)		-.0001 (.002) [p = .966]

### Sports

Table 4a shows that there is no relationship between the percentage of green space in the living environment and whether or not people participate in sports activities and the number of minutes people spend on sports activities. People with more green space in their living environment do not participate more often in sports activities and do not spend more minutes on sports activities.

**Table 4:** 

a: Multivariate regression analysis for the influence of the percentage of green space on sports and walking and cycling during leisure time: parameter and standard error [p-value]						
	**Sports (n = 4.899)**		**Walking during Leisure time (n = 4.899)**		**Cycling during leisure time (n = 4.899)**	
			
	**1 km**	**3 km**	**1 km**	**3 km**	**1 km**	**3 km**
	
**Physically active (yes/no)**						
Percentage of green (1 km)	.002 (.002) [p = .255]		-.007 (.002) [p < .001]		-.006 (.002) [p < .001]	
Percentage of green (3 km)		.003 (.002) [p = .234]		-.006 (.002) [p = .009]		-.0004 (.003) [p = .887]
						
**Minutes of activity per week (calculated for those who are physically active)**						
Percentage of green (1 km)	.14 (.3) [p = .582]		-.24 (.24) [p = .317]		-.3 (.2) [p = .145]	
Percentage of green (3 km)		-.05 (.4) [p = .897]		-.98 (.32)** [p = .002]		-.3 (.3) [p = .204]

*Note *All analyses are controlled for age, gender, level of education, income and urbanicity.						


b: Multivariate regression analysis for the influence of the percentage of green space on walking and cycling for commuting purposes and gardening: parameter and standard error [p-value]						

	**Walking for commuting purposes (n = 2.816)**		**Cycling for commuting purposes (n = 2.816)**		**Gardening (n = 3.951)**	
			
	**1 km**	**3 km**	**1 km**	**3 km**	**1 km**	**3 km**
	
**Physically active (yes/no)**						
Percentage of green (1 km)	.002 (.004) [p = .5912]		-.005 (.002) [p = .032]		.008 (.002) [p < .001]	
Percentage of green (3 km)		-.001 (.005) [p = .844]		-.007 (.004) [p = .110]		.005 (.003) [p = .062]

**Minutes of activity per week (calculated for those who are physically active)**						
Percentage of green (1 km)	.9 (.5) [p = .086]		.83 (.2) [p < .001]		1.4 (.3) [p < .001]	
Percentage of green (3 km)		.4 (.7) [p = .622]		.62 (.25)* [p = .014]		1.45 (.45) [p = .001]

*Note *All analyses are controlled for age, gender, level of education, income and urbanicity.						

### Walking during leisure time

With regard to walking during leisure time, the results show that people walk less often during leisure time when there is more green space in their direct living environment. This relationship is as large in a 1-km radius as in a 3-km radius around one's home (Table 4a). Our analysis also shows that people spend less leisure time on walking when there is more green space in a 3-km radius around their home. People with 20% green space in a 3-km radius around their home walked approximately 250 minutes per week for leisure, whereas people with 80% green space in a 3-km radius around their home walked approximately 190 minutes per week during leisure time.

### Cycling during leisure time

There is also a negative relationship between the percentage of green space in the living environment and whether or not people cycle during leisure time (Table 4a). This negative relationship is only significant for the percentage of green space in a 1-km radius around one's home. There is no significant relationship between the percentage of green space in the living environment and the time people spend on cycling during leisure time.

### Walking for commuting purposes

There is no significant relationship between the percentage of green space and walking for commuting purposes (table 4b). People with more green space in their living environment do not walk more often for commuting purposes and do not walk for commuting purposes for a longer period.

### Cycling for commuting purposes

With regard to cycling for commuting purposes, our results show that there is a negative relationship between the percentage of green space in a 1-km radius and whether or not people cycled for commuting purposes (Table 4b). However, if people cycled for commuting purposes they were likely to spend more time on it if they had a higher percentage of green space in a 1-km and 3-km radius around their homes. People with 20% green space in a 1-km radius around their home cycle approximately 120 minutes per week for commuting purposes, whereas people with 80% green space in a 1-km radius around their home cycle approximately 170 minutes per week for commuting purposes.

### Gardening

Table 4b shows the results for the analysis of the relationship between the percentage of green space and gardening. People with a higher percentage of green space in a 1-km radius around their home garden more often. The figures in Table 4b show that only about 40% of people with 20% green space in a 1-km radius around their home are active in gardening, whereas this is true for a mere of about 50% of those who have 80% green space in a 1-km radius around their home. Furthermore, people who garden spend more time on gardening when they have more green space in a 1-km or 3-km radius around their home. People with 20% green space in a 1-km radius around the home garden approximately 180 minutes per week, whereas people with 80% green space in a 1-km radius around their home garden 265 minutes per week.

For the types of physical activity for which a relation with green space was found (walking during leisure time, cycling during leisure time, cycling for commuting purposes and gardening) we analysed whether the relationship between green space and the type of physical activity differed for the type of green space, level of urbanicity, age group and socio-economic group (operationalised as education or income subgroups). In the next sections the general results of these analyses are given.

### Type of green space

To investigate which type of green space especially promotes physical activity we analysed the relation for different types of green space, namely agricultural, natural and urban green space. Overall, the analyses show that the relationship between agricultural green space and the different types of physical activity was strongest.

### Urbanicity

To investigate whether the relationship between green space and the level of physical activity differs by urbanicity we analysed the relation in the different levels of urbanicity. The analyses show that in the more rural areas the relationship between green space and physical activity is stronger than in the more urban areas. The relationship between green space and physical activity is strongest in slightly urban areas.

### Age groups

To test our hypothesis that the relationship between green space and physical activity is stronger for youth and elderly we performed subgroup analyses for different age groups (the analyses were controlled for age, gender, level of education, income and urbanicity). These analyses show that the relationship between green space and physical activity for different age groups differs per type of activity. The negative relationship between the percentage of green space and whether or not people walk during leisure time was strongest for people aged between 12 and 25 years, followed by elderly and the negative relation was least strong for adults aged between 26 and 65. Concerning whether or not people cycle during leisure time, the negative relationship was strongest for children. With regard to cycling for commuting purposes the analyses show that the older people are, the stronger the relation. For gardening, the relation was strongest for elderly and people aged between 17 and 25.

### Socio-economic groups

We hypothesized that the relationship between green space and physical activity is stronger for people with a lower socio-economic status. Our results indicate (not shown in table) that the relationship between the percentage of green space in the living environment and the types of physical activity was stronger for people with a lower level of education and people with a lower income.

### Physical activity as an explanation for the relationship between green space and health

Only for the number of minutes spent on cycling for commuting purposes and for the frequency and duration of gardening a significant positive relationship is found with the percentage of green space in the living environment. Therefore we only investigated whether these kinds of physical activity can explain (part of) the relationship between green space and health.

Table 5 (model 1a and 1b) shows that adding the number of minutes people spend on cycling for commuting purposes does not have any effect on the significant influence of green space on self-perceived health. The relation between green space and health does not diminish when minutes spent on cycling is added to the model. There was no relation between health and the percentage of green space in a 3-km radius around people's home for people who cycled to their work (model 2). Apparently, green space in a 3-km radius around people's home does not influence health for this subgroup of people.

**Table 5:** Multilevel logistic regression analysis for perceived general health for people who cycle for commuting purposes (n = 1.153): parameter and standard error [p-value]

	**Perceived general health ('excellent/very good/good' = 1)**		
	**Model 1a**	**Model 1b**	**Model 2**

% of green (1 km)	.010 (.003) [p = .002]	.011 (.003) [p = .005]	
% of green (3 km)			.006 (.005) [p = .194]
			
Time spent on cycling for commuting purposes (minutes)		-.001 (.001) [p = .095]	

*Note *All analyses are controlled for age, gender, level of education, income and urbanicity.

Table 6 shows that there is a significant relation between whether or not people garden and the self-perceived health of people. People who garden feel healthier. However, whether or not people garden cannot explain the relation between green space and health, because adding this variable to the model (model 1b) does not have any effect on the relationship between green space and health.

**Table 6:** Multilevel logistic regression analysis of gardening activity (yes/no) (n = 3.942) for perceived general health: parameter and standard error [p-value]

	**Perceived general health ('excellent/very good/good' = 1)**			
	**Model 1a**	**Model 1b**	**Model 2a**	**Model 2b**

% of green (1 km)	.006 (.002) [p = .003]	.005 (.002) [p = .005]		
% of green (3 km)			.006 (.003) [p = .021]	.006 (.003) [p = .026]
				
Gardening activity (yes = 1)		.195 (.073) [p = .008]		.203 (.073) [p = .005]

*Note *All analyses are controlled for age, gender, level of education, income and urbanicity.

Table 7 shows that there is no significant relation between health and the percentage of green space for the subgroup of people who garden. Furthermore, this table shows that the number of minutes spent on gardening is not related to perceived general health.

**Table 7:** Multilevel logistic regression analysis of people who spend time on gardening (n = 1.877) for perceived general health: parameter and standard error [p-value]

	**Perceived general health ('excellent/very good/good' = 1)**			
	**Model 1a**	**Model 1b**	**Model 2a**	**Model 2b**

% of green (1 km)	.005 (.003) [p = .062]	.005 (.003) [p = .062]		
% of green (3 km)			.006 (.004) [p = .106]	.006 (.004) [p = .103]
				
Time spent on gardening (minutes)		.000 (.000) [p = .992]		.000 (.000) [p = .949]

*Note *All analyses are controlled for age, gender, level of education, income and urbanicity.

## Discussion

### Green space and physical activity

Results from this study suggest that the amount of green space in people's living environment has little influence on people's level of physical activity. No significant relations were found between the percentage of green space in the living environment and whether or not people meet the Dutch public health recommendations for physical activity, sports and walking for commuting purposes.

We found a negative relation between the amount of green space and walking and cycling during leisure time. People in greener living environments undertake these activities less often. These results are in accordance with the Dutch study by Den Hertog et al. (2006) [25] in which different neighbourhoods in the city of Amsterdam were compared and in which a negative relation between green space and walking and cycling was found as well.

The finding that people with more green space in their living environment less often walk or cycle is probably due to the fact that in greener living environments, facilities such as shops are further away and people more often use a car to reach facilities. Furthermore, greener living environments in more urban areas are often set out more spaciously, reducing the facility density and increasing the possibility of parking a car near one's home. The study by Den Hertog et al. (2006) [25] performed in the Netherlands showed that – within an urban environment – both the density of facilities and parking possibilities were important determinants for the amount of physical activity undertaken, especially walking and cycling. In neighbourhoods with a high density of facilities and without private parking spaces, people more often choose to walk or cycle [25].

Our results concerning walking and cycling during leisure time contrast with studies which find rather strong indications for a relationship between attractive streetscapes and the amount of walking and cycling in Australia, the United States and the U.K. [9]. A reason for the differences found could be that our data on green space did not provide specific information on the attractiveness of the streetscape. We were not able to investigate the influence of small green areas, like for instance trees along the roads. Furthermore, the differences found could be due to the walking and cycling culture in the Netherlands, which gives citizens of the Netherlands lots of opportunities to walk and cycle safely elsewhere, even when there is no green space in the direct vicinity of their homes.

We did find a positive relation between green space and gardening and cycling for commuting purposes. Especially the amount of agricultural green space influenced these types of physical activity positively. People with more agricultural green space in their living environment garden more often and for a longer duration, and if they cycle for commuting purposes, they spend more time on it. The fact that people with more agricultural green space in their living environment garden more often and spend more time on it, is most probably due to the fact that people in areas with more agricultural green space own larger size gardens.

An explanation for the fact that people who cycle for commuting purposes spend more time on this – a result which was also found in the study of Wendel-Vos et al. (2004) [24] – is that living environments with more agricultural green space are often located further away from cities, which is where most jobs are available. Therefore, people in the areas with more agricultural green space have to cycle more minutes to reach their work or school. Or as Wendel-Vos et al. (2004) [24] explains, 'the result reflects the fact that people in outskirts of town spend more time on bicycling to the city'.

Regarding the relationship between green space and physical activity in different levels of urbanicity, the relation appeared to be stronger in the more rural areas than in the urban areas. The strongest relation was found in slightly urban areas.

Concerning the subgroup analysis, the link between physical activity and green space was strongest for people aged under 25 and for elderly, lower educated people and people with a low income. This is in line with our hypothesis that children, elderly and lower socio-economic groups, spend more time in the vicinity of their homes and are therefore likely to be more affected by the design of their direct living environment.

### Physical activity as an explanation for the relationship between green space and health

The fact that people spend more time on cycling for commuting purposes and on gardening could not explain the relation between green space and health. Therefore, we can conclude that physical activity is not a likely mechanism behind the relation between green space in people's direct living environment and health that was found in previous studies.

In analyses in which only people were included who garden or people who spend time on cycling for commuting purposes, no relationship was found between green space and self-perceived health. This can be explained by the fact that in these small subgroups the variation in green space is smaller. Additionally, people who spend time on gardening already spend time in green space and the extra benefit of green space outside their homes might not be discernable. Furthermore, people who garden and people who cycle for commuting purposes are probably healthier.

However, it is important to note that although people with greener living environments do not more often meet the Dutch public health recommendations for physical activity, it is possible that they more often undertake physical activity in a green environment. Different studies have shown that people with more green space in their living environment more often use green space [40]. Because we did not have any data on *where *people were physically active, we were not able to find out whether people with greener living environments more often exercise *in green spaces*. A study by De Vries et al. (2004) [22] showed that the local green space supply does not determine *how often *people engage in recreation, but it does determine *where *people engage in recreation. The findings of this study also suggest that if there is no green space available people seek alternatives in other environments. Undertaking more physical activity in a green environment as opposed to an urban environment could have health benefits in the form of reduced stress symptoms [41,42].

Furthermore, it is possible that the lack of an relation between the level of physical activity and the amount of green space is due to the high density of sports facilities and safe cycle tracks and footpaths almost anywhere in the Netherlands. Under these circumstances, the availability of green space is not a necessary condition to be physically active.

### Strengths and limitations

This is one of the first studies to investigate whether the amount of physical activity undertaken can contribute to the explanation for the relation between green space and health found in previous studies. Where most studies only investigate the relationship between physical activity and green space or the relationship between green space and health, we investigated both the relationship between physical activity and green space as well as the relationship between green space, physical activity and health. Furthermore, unlike other studies performed in the Netherlands or in other countries, this study specifically investigates the relation between different types of green space and different types of physical activity for different subgroups and levels of urbanicity.

The data on health (and physical activity) and land use were derived from various databases; consequently, there is no single source bias.

In our study we used objective environmental measures. Objective environmental measures reduce the risks of respondent bias. However, subjective environmental measures can also provide important information. People's perception of green spaces may, in fact, motivate their behaviour more than the actual amount of available green space. Green spaces or green spaces that are considered unsafe or of poor quality tend to be avoided. Thus, supplementing objective measures with measures of an individual's perception will improve our understanding of how the green environment affects physical activity level. We used a self-report measure for physical activity which is the most commonly used measure for assessing physical activity [7,33]. Using a self-report measure for physical activity has the advantage that it is easy to administer and generally acceptable to participants, and can measure a wide range of values [33]. Self-report measures have the disadvantage of incomplete recall and exaggeration of the amount of activity [7]. For this study there are no direct consequences in this respect, because we are interested in the relationship between green space and physical activity and it is not likely that people living in greener living environments will exaggerate more or less than people in less green living environments.

The measure used for physical activity, the SQUASH questionnaire, was not validated for each of the specific physical activities which are distinguished in this paper.

A limitation of our study is its cross-sectional design. The study does not inform us about the direction of causation. A second limitation is that we did not know where people were physically active. Other studies have found a significant correlation between the availability of green space and the use of green space [40]. Our study shows that the absence of green space does not necessarily lead to less physical activity in general, but that people probably compensate for the lack of green space by being physically active elsewhere. Future research should include questions on *where *people are physically active.

Furthermore, some potentially important control variables could not be taken into account. It would, for instance, have been interesting to see whether the density of (sports) facilities in different living environments has an effect on the level of physical activity. In addition, ownership of a dog, which has been proved to influence the level of physical activity, could not be taken into account [43]. Research has shown that there are rather strong indications for a relation between attractive streetscapes and the amount of walking and cycling in Australia, United States and U.K. [9]. Unfortunately, we were not able to investigate whether this relationship can also be found in the Netherlands, because we did not have detailed information on the greenness of the streetscape.

## Conclusion

This study indicates that the amount of green space in the living environment is related to the overall level of physical activity only to a very limited extent. Furthermore, our study indicates that the amount of physical activity among people who live in greener environments cannot explain the relation between green space and health that was found in previous studies.

## Competing interests

The authors declare that they have no competing interests.

## Authors' contributions

All authors have made a significant contribution to the reported paper. JM was the main author of the manuscript and was involved in all aspects of this paper. RV and PG contributed significantly to the conception and design of the study and the interpretation of the results. PS helped with the design and the interpretation of the statistical analysis. Besides the authors, no others have participated in any aspects of the research project. All co-authors have seen and approved the final version of the paper and have agreed to its submission for publication.

## Pre-publication history

The pre-publication history for this paper can be accessed here:


